# Morphometric Study of the Mastoid Triangle for Sexual Dimorphism of Dry Skulls in the North Indian Population

**DOI:** 10.7759/cureus.14859

**Published:** 2021-05-05

**Authors:** Jigyasa Passey, Suniti Pandey, Rahul Singh, Shailendra Singh

**Affiliations:** 1 Department of Anatomy, Hamdard Institute of Medical Sciences & Research, New Delhi, IND; 2 Department of Anatomy, Ganesh Shankar Vidyarthi Memorial (GSVM) Medical College, Kanpur, IND; 3 Department of Anatomy, Integral Institute of Medical Sciences & Research (IIMSR), Lucknow, IND

**Keywords:** mastoid triangle, sexual dimorphism, dry skulls

## Abstract

The mastoid process is a conical projection from the undersurface of the temporal bone. Examination of skeletal remains by anthropologists requires sex determination. The present study proposes to determine sex from morphometry of the mastoid process. The study was conducted on 300 dried skulls with the help of a digital vernier caliper. Discriminant functional analysis was performed. The parameters measured were the mastoid triangle, which is formed by specific points the porion, mastoidale, and asterion, and the linear distances between them. All parameters were higher in male skulls with a high level of significance. The area of the mastoid triangle proved to be the best parameter for sex discrimination.

## Introduction

The mastoid process is a conical projection lying in the posterior region of the temporal bone. The temporal bone is a paired cranial bone that has four parts: squamous part, tympanic part, styloid process, and petromastoid part. The petro mastoid part is further divided into the petrous and mastoid parts. The mastoid process has an outer surface roughened by attachments of the occipitofrontalis and auricularis posterior and a lateral surface where the sternocleidomastoid, splenius capitis, and longissimus capitis are attached. For many anthropologists, while excavating skeletal remains or in cases of unforeseen disasters, identification of gender is the preliminary task. Petaros et al. [1] and Kruger et al. [2] stated that the mastoid process is one of the most sexually dimorphic features in the human skull and is, therefore, often used to identify the sex of skeletons. A major role in the gender identification of skeletal remains may be played by morphometric osteological criteria and lays the foundation for full identification. In the study by Kruger et al. [2], the morphological methods have not been addressed accurately. Sex is best assessed from the pelvis but it is, very often, damaged. Therefore, individual parts of the skull like the mastoid process are being analyzed for sex determination due to their anatomically advantageous placement in the skull.

## Materials and methods

**Figure 1:**
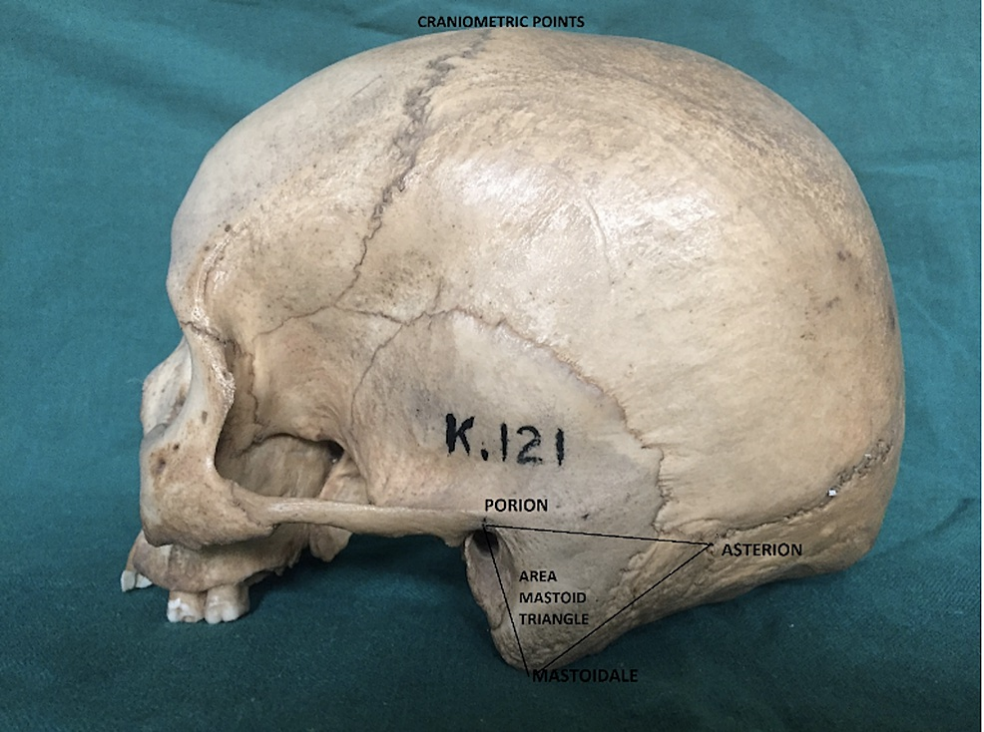
The three craniometric points, i.e., porion, asterion, and mastoidale, and the mastoid triangle formed

The present study was conducted on 300 dry skulls of known sex, 190 male and 110 females, in the anthropology museum of our department. The skulls were sexed on appearance according to the criteria specified by Williams and Rogers [3] as specified in Table 1. The materials employed were dry skulls, a digital vernier caliper (precision 0.01 cm), and a digital camera for illustration. The inclusion criteria were all the adult dry skulls of both sexes without destruction of the mastoid bone in the region of the craniometric points. The exclusion criteria were all skulls with an atrophied, decomposed, and deformed mastoid process and juvenile and senile skulls. The three craniometric points were identified in dry skulls, the porion (Po), which is the superior-most point of the external acoustic meatus, the mastoidale (Ma), which is the inferior-most point of the mastoid process, and the asterion (As), which is the meeting point of the three posterior skull sutures, i.e., lambdoid, occipitomastoid, and parietomastoid. These craniometric points were selected and marked by a single investigator to avoid an intraobserver error. A triangle was made using these three points on the skulls and the measurements were carried out by a digital vernier caliper as shown in Figure 1. The mastoid triangle area was calculated using Heron's formula.

**Table 1:** The sex of the skulls was established on the basis of gross appearance by comparing the following external features Williams and Rogers [3]

No	Male Characteristics	Female Characteristics
1	Supraorbital ridges are more prominent	Supraorbital ridges are less prominent
2	Glabella is more prominent	Glabella is less prominent
3	Frontal eminences are more prominent	Frontal eminences are less prominent
4	Upper orbital margins are blunt	Upper orbital margins are sharper

Basic statistics and discriminant functional analysis

After using a p-value with the level of significance set at p < 0.05, an independent sample t-test was performed to test the significance of all the variables between both sexes to distinguish between the male and female mean values for each variable. Then the direct discriminant functional analysis was performed in which low values of Wilks lambda, high values of Eigenvalue, the Canonical correlation, and percentage of correct prediction accuracy denote higher prediction. All variables were entered into a stepwise discriminant function analysis.

## Results

All the parameters measured, i.e., the porion-mastoidale, mastoidale-asterion, asterion-porion distance, and the area of the mastoid triangle proved to have a higher value in males as compared to females and the differences were statistically significant for all these parameters. According to Table 2, the porion-mastoidale distance was computed as 31.69 ± 1.78 mm and 30.62 ± 2.08 mm on the right and left sides, respectively, for male skulls. The values of female skulls were 30.11 ± 3.17 mm and 28.91 ± 3.11 mm on the right and left sides. The mastoidale-asterion distance was calculated as 48.93 ± 2.06 mm and 48.39 ± 1.03 mm on the right and left sides for male skulls, whereas, for females, the values were 45.03 ± 3.07 mm and 45.19 ± 2.93 mm on the right and left sides, respectively. The asterion-porion distance was calculated as 46.97 ± 2.51 mm on the right side and 47.1 +/- 3.18 mm on the left side for males, and 44.00 +/- 1.24 mm on the right side and 45.17 +/- 1.08 mm on the left side for females. Finally, the area of the mastoid triangle was calculated to be 650.29 ± 50.45 mm^2^ on the right side and 648.97 ± 9.88 mm^2^ on the left side for males, and 590.61 ± 47.7 mm^2^ on the right side and 582.19 ± 12.57 mm^2^ on the left side for females. The right-left side differences were statistically insignificant for these parameters.

**Table 2:** Measurements obtained on dry skulls M-Male, F-Female, R-Right, L-Left

S.no	Parameter	Gender	Mean ± SD		Mean ±SD		P-value	Significance
			R		L			
1.	Porion- mastoidale (mm)	M	31.69	1.78	30.62	2.08	<0.0001	Highly Significant
		F	30.11	3.17	28.91	3.11		
2.	Mastoidale asterion (mm)	M	48.93	2.06	48.39	1.03	0.001	Significant
		F	45.03	3.07	45.19	2.93		
3.	Asterion-porion (mm)	M	46.97	2.51	47.10	3.18	0.001	Significant
		F	44.00	1.24	45.17	1.08		
4.	Area of mastoid triangle (mm)	M	650.29	50.45	648.97	9.88	<0.0001	Highly Significant
		F	590.61	47.7	582.19	12.57		

The discriminant functions were calculated for each variable. The best function in the present study is obtained by the area of the mastoid triangle, which shows the lowest Wilk’s lambda (0.655), the highest Eigenvalue (0.865), the highest canonical correlation (0.501), and the highest percentage of correct classification (78%) (Table 3). The second best function is obtained by the porion-mastoidale length. On the other hand, the function produced by the mastoidale-asterion distance shows far less discriminative capacity, as the function includes the highest Wilk’s lambda (0.750), the lowest Eigenvalue (0.117), the lowest canonical correlation (0.301), and the lowest percentage of correct classification (69%). The objective of discriminant analysis is to rank the variables according to their contribution to the separation of two groups. Exploring the data showed that the area of the mastoid triangle was the best predictor for sex determination followed by the porion-mastoidale length (Table 4). Also, the mastoidale-asterion distance contributes the least to sex determination.

**Table 3:** Variable wise calculation of discriminant functions on dry skulls M-Male, F-Female, T-Total

S.no	Variable	Wilks lambda	Eigenvalue	Canonical correlation	Prediction accuracy		
					M	F	T
1.	Mastoidale-asterion	0.750	0.117	0.301	70%	68%	69%
2.	Porion-mastoidale	0.687	0.989	0.422	76%	75%	75.5%
3.	Asterion-porion	0.692	0.401	0.326	70%	70%	70%
4.	Area of mastoid triangle	0.655	0.865	0.501	100%	80%	78%

**Table 4:** Ranking of variables in determining the sex of dry skulls

S. no	Variable	Rank	Percentage prediction accuracy
1.	Area of mastoid triangle	1	78%
2.	Porion-mastoidale	2	75.5%
3.	Asterion-porion	3	70%
4.	Mastoidale-asterion	4	69%

## Discussion

Analysis of the characteristics of the mastoid process is important in the determination of sex for forensic purposes and anthropologists. Our study showed that the dry skulls can be correctly classified into male and female by using a metric analysis of the mastoid triangle. Madadin et al. [4] stated that the demographic assessment of skeletal remains in forensic investigations includes the identification of sex. Jung et al. [5] stated that the conventional methods for evaluating the mastoid as a sex indicator cannot determine whether the mastoid shape is an independent and reliable sex indicator. Sinhorini et al. [6] and other researchers also stated that the pelvis and skull are the regions of the human skeleton that most clearly show sexual dimorphism, with the pelvis being superior to the skull for sex estimation owing to reproductive and hormonal factors. However, as many skeletons are found incomplete, it is important to be able to determine sex from analyzing the skull as well as the pelvis. De Paiva et al. [7], Kemkes and Gobel [8], and Sumati et al. [9] also stated that the qualitative aspects of the mastoid process, such as their size and muscular impressions, are very good indicators of sexual dimorphism. However, quantitatively, the parameters employed remain inconclusive. Mahakkanukrauh et al. [10] and many authors suggested that the morphological study was more subjective than the metric. There are various craniometric studies in different populations. The compact structure and anatomical position of the mastoid region of the skull make it highly resistant to any physical damage and thus it may remain intact in otherwise damaged and fragmented skulls. The dimorphic variations of gender develop during intrauterine life and later manifest as differences in bone weight, length, size, and mineral density. Males have both longer and more intense growth bouts than females, therefore, this extended growth pattern creates a difference in size, classically seen in the skull, where the growth spurts affect most structures. The secondary sexual changes are influenced by hormones, which play a role in the development of the musculoskeletal system. The unequal mastoid process is formed due to pneumatization (air-filled cavity), and the size of the mastoid air cell system is determined by the degree of pathological involvement of the middle ear during childhood. Descriptive statistics demonstrate patterns of sexual dimorphism in the mastoid region, and the results indicate that a quantitative approach provides greater consistency in identification than the qualitative characterization of the mastoid region, as it is used almost exclusively in current practice. The discriminant functional analysis provides sex-assessment criteria with regards to human skeletal remains; moreover, it is objective and simple. Its discriminatory effectiveness is more even with the minimum number of traits. Careful determination of the metric parameters forms a cornerstone element of this type of analysis because of difficulty in intra-observer repeatability and intra-observer reproducibility according to Nagaoka et al. [11]. Yet another problem is that the efficacy of the sex discriminant function is not sure in populations other than ones from which they have been derived. Paiva et al. [7] introduced an easy technique for sex determination, starting from the temporal bone, with a small observational error and with high predictability degree. The technique is based on the triangular area calculation obtained between the points porion, mastoidale, and asterion measured from a xerographic copy of skulls. Many researchers have conducted studies on metrical assessment of the mastoid process for sexual dimorphism, however, to the best of our knowledge, a sample size of three dry skulls has not been studied. Our study elicited that all parameters were higher in males as compared to females and were similar to other North Indian studies indicating ethnic variation. However, as per Kanchan et al. [12], it may be noted that the mastoid triangle as an indicator of sex is of limited significance without population reference, further strengthening our claim on ethnic inclination. Using discriminant functional analysis, it was revealed that the area of the mastoid triangle was the best predictor for sex determination. The limitation of this study is that it was conducted on only North Indian skulls, whereas skulls of other regions can also be explored. Furthermore, measurements performed on digital X-ray films and CT scans may also be done to further check the validation of the mastoid triangle as a tool for sex determination. Nevertheless, the results of the present study are encouraging for the sex determination of dry skulls while employing a metric analysis of the mastoid triangle. According to Table 5, we calculated the porion-mastoidale distance, which was computed as 31.69+/-1.78 mm and 30.62+/-2.08 mm on the right and left sides, respectively, for male skulls. Also, 30.11 +/- 3.17 mm and 28.91 +/- 3.11 mm on the right and left sides of females, respectively, for female skulls. In comparison with other researchers, our data proved to be similar to Vidya et al. [13], Gangrade et al. [14], and Sivakumar et al. [15]. Our results were closest to the study of Gangrade et al. [14] who studied skulls in Nagpur and Udaipur and got 31.53 ± 3.20 mm on the right side and 30.48 ± 3.56 mm on the left side for males, and 28.47 ± 2.16 mm on the right side and 28.28 ± 2.31 mm on the left side for females. Our results were marginally dissimilar to the study of Gangrade et al. [14] who studied skulls from Nagpur and Udaipur and got 52.39 ± 4.20 mm on the right side and 52.48 ± 5.56 mm on the left side for males and 49.07 ± 2.16 mm on the right side and 48.51 ± 2.31 mm on the left side for female skulls, which were higher than our study. Her work coincided with that of Suazo et al. [16] for the mastoidale-asterion distance (Table 6). The asterion-porion distance, as per Table 7, was calculated as 46.97 ± 2.51 mm on the right side, 47.1 +/- 3.18 mm on the left side for males, and 44.00 +/- 1.24 mm on the right side and 45.17 +/- 1.08 mm on the left side for females. This is in agreement with results obtained by Saini et al. [17] who studied the North Indian population. In comparison with other researchers, our analysis was fairly similar to Bhagya et al. [18]. Our results were marginally dissimilar to the study by Gangrade et al. [14] who studied skulls from Nagpur and Udaipur. Sivakumar et al. observed results slightly higher than ours. In Table 8, the area of the mastoid triangle was calculated to be 650.29 mm^2^ ± 50.45 in males and 590.61 mm^2^ ± 47.7 in females. This is in agreement with results obtained by Saini et al. [17] who conducted studies on the North Indian population. In comparison with other researchers, our analysis was similar to that of Shah et al. [19]. Our results were marginally dissimilar to the study by Gangrade et al. [14], Sivakumar et al. [15], and Bhagya et al. [18] and obtained results higher than our study. Table 9 depicts the overall ranking of the parameters according to their sex differentiating power, i.e., the area of the mastoid triangle was the best predictor of sex while the mastoid-asterion was the least. The mastoid process can thus be used as a marker of sex as well as ancestry of individuals and populations as stated by Passey J, Mishra RS, Singh R et al. [20]. On comparing with the results of other studies, the present study shows that the dimensions of the mastoid process measured by the anthropometric technique can be accountable in medicolegal investigations, and it can be taken as a sex indicator among North Indians.

**Table 5:** Morphometric comparison of the porion-mastoidale distance between different researchers on dry skulls M-Male, F-Female, R-Right, L-Left

S.No	Researcher	Race/Region	M (mean+/-SD)		F (mean+/-SD)		
			R (mm)	L (mm)	R (mm)		L (mm)
1.	Suazo et al [16]	Brazilian	30.72+/-2.73	29.22+/-2.73	27.55+/-2.78		29.74+/-4.14
2.	Sumati et al [9]	North Indian	28.3+/-4.04		23.18+/-4.24		
3.	Saini et al [17]	North Indian	31.77+/-3.07		27.98+/-3.47		
4.	Vidya et al [13]	South Indian	35.3+/-0.42	34.2+/-0.30	35.4+/-0.42	33.6+/-0.34	
5.	Gangrade et al [14]	Nagpur And Udaipur	31.53+/-3.20	30.48+/-3.56	28.47+/-2.16	28.28+/-2.31	
6.	Bhagya et al [18]	South Indian	29.52+/-3.34		24.26+/-3.77		
7.	Sivakumar et al [15]	South Indian	31.2+/-3.4		28.65+/-3.1		
8.	Present Study	North Indian	31.69+/-1.78	30.62+/-2.08	30.11+/-3.17	28.91+/- 3.11	

**Table 6:** Morphometric comparison of the mastoidale-asterion distance between different researchers on dry skulls M-Male, F-Female, R-Right, L-Left

S. No	Researcher	Race/Region	M (Mean+/- SD)			F (Mean+/- SD)		
			R (mm)		L (mm)	R(mm)		L (mm)
1.	Suazo et al [16]	Brazilian	50.21+/-4.96		50.22+/-4.95	48.35+/-3.8		50.17+/-5.1
2.	Saini et al [17]	North Indian	47.83+/-4.06			43.00+/-4.32		
3.	Gangrade et al [14]	Nagpur And Udaipur	52.39+/-4.32	52.4+/-5.46		49.06+/-3.02	48.51+/-3.27	
4.	Bhagya et al [18]	Nagpur And Udaipur	50.11+/-4.54			46.51+/-4.12		
5.	Sivakumar et al [15]	South Indian	50.0+/-5.0			47.6+/-5.1		
6.	Shubhangi et al [21]	Maharashtra	49.02+/-2.94	49.03+/-3.24		45.77+/-3.95	45.27+/-3.61	
7.	Present Study	North Indian	48.93+/-2.06	48.39+/-1.03		45.03+/-3.07	45.19+/-2.93	

**Table 7:** Morphometric comparison of the asterion-porion distance between different researchers on dry skulls M-Male, F-Female, R-Right, L-Left

S.No	Researcher	Race/Region	M (mean+/-SD)		F (mean+/-SD)	
			R (mm)	L (mm)	R (mm)	L(mm)
1.	Nagaoka et al [11]	Japanese	49.3+/-2.64		47.0+/-2.9	
2.	Suazo et al [16]	Brazilian	47.45+/-4.43	47.51+/-3.4	46.74+/-3.30	47.53+/-3.80
3.	Saini et al [17]	North Indian	47.89+/-3.17		44.69+/-3.75	
4.	Gangrade et al [14]	Nagpur And Udaipur	49.02+/-4.076	49.25+/-3.27	46.98+/-2.96	46.59+/-2.88
5.	Bhagya et al [18]	South Indian	44.48+/-4.14		42.87+/-3.08	
6.	Sivakumar et al [15]	South Indian	48.15+/-3.6		45.7+/-3.3	
7.	Shubhangi et al [21]	Maharashtra	41.19+/-3.29	41.42+/-3.6	39.19+/-3.54	39.25+/-3.46
8.	Present Study	North Indian	46.97+/-2.51	47.1+/-3.18	44.00+/-1.24	45.17+/-1.08

**Table 8:** Morphometric comparison of the areas of the mastoid triangle formed by craniometric points: porion-mastoidale, mastoidale-asterion, and asterion-porion by different researchers on dry skulls M-Male, F-Female, R-Right, L-Left

Researcher	Region	M (mm^2^)		F (mm^2^)	
		R	L	R	L
De Paiva et al [7]	Brazil	752.16	753.22	608.70	602.54
Suazo et al [16]	Brazil	703.34	674.45	624.08	685.64
Gangrade et al [14]	Nagpur Udaipur	749.6	726.1	650.3	638.6
Shah et al [19]	Gujrat	663.66	662.54	595.92	589.29
Sivakumar et al [15]	South India	718.25		632.7	
Present Study	Uttar Pradesh	650.29	648.97	590.61	582.19

**Table 9:** Comparison of the sex prediction accuracy of variables by different authors on dry skulls

S. no.	Variable	Saini et al [17]	Present study
1.	Porion-mastoidale length	69.9	75%
3.	Area mastoid triangle	---	78%
5.	Mastoidale asterion	75.4	69%
8.	Asterion- porion	72.5	70%

## Conclusions

The results of this study are encouraging, offering a better opportunity to identify sex using the mastoid process. In the present study, sex determination using mastoid parameters was established for the North Indian population when either a fragmented skull is obtained or when only the mastoid region is obtained in isolation. It was concluded that the mean mastoid triangle was more in male skulls than female skulls irrespective of race or region.
